# Effect of navigated transcranial magnetic stimulation for glioma surgery outcomes: a systematic review and meta-analysis

**DOI:** 10.1515/med-2025-1326

**Published:** 2026-07-02

**Authors:** Zhipeng Xu, Jinrong Wang, Wenming Wang, Hua Liu

**Affiliations:** Department of Neurosurgery, Kunshan Hospital of Traditional Chinese Medicine, Kunshan Affiliated Hospital of Yangzhou University, Kunshan, P.R. China; Kunshan Key Laboratory of Integrative Medicine for the Treatment of Exercise and Neurological Diseases, Kunshan, P.R. China; Department of Neurosurgery, Affiliated Kunshan Hospital of Jiangsu University, Kunshan, Jiangsu, P.R. China; Kunshan Biomedical Big Data Innovation Application Laboratory, Kunshan, Jiangsu, P.R. China

**Keywords:** navigated transcranial magnetic stimulation, glioma, meta-analysis, extent of resection, preoperative planning

## Abstract

**Objectives:**

Gliomas are the most common primary brain tumors in adults, often linked to poor prognosis despite advances in treatment. Navigated transcranial magnetic stimulation (nTMS) is a non-invasive preoperative mapping technique that may improve glioma surgery outcomes by enhancing tumor resection and preserving neurological function. This meta-analysis evaluates the impact of nTMS on glioma resection outcomes.

**Methods:**

A literature search was conducted in PubMed, Embase, Cochrane Library, and Web of Science up to September 2024 for cohort or case-control studies comparing nTMS-guided surgery with conventional planning, with or without subcortical fibre-guided tractography (SFG) and intra-operative neurophysiological monitoring (IONM). Data on extent of resection, motor function, quality of life (Karnofsky Performance Status), and complications were extracted. Statistical analysis was performed using RevMan 5.3. This study was registered at PROSPERO (registration number CRD42025641788).

**Results:**

Five studies with 736 patients were included. The nTMS group showed significantly higher rates of gross total resection (GTR) and lower rates of subtotal resection (STR) compared to the non-nTMS group. No significant differences were found in postoperative motor function or quality of life. Two studies comparing nTMS + SFG + IONM with IONM alone demonstrated higher GTR rates (absolute increase 12–18 %) and reduced permanent motor paresis (3–6 % vs. 9–14 %); one reached statistical significance for neurological outcomes.

**Conclusions:**

Pre-operative nTMS significantly improves the anatomical radicality of glioma resection without increasing early neurological morbidity. Integration of nTMS with SFG-based tractography and IONM may confer additional functional protection, highlighting the value of a multimodal mapping strategy for tumors abutting motor-eloquent cortex. Further studies are needed to confirm these findings.

## Introduction

Gliomas are the most common primary brain tumors, arising from glial or precursor cells and accounting for approximately 23 % of all primary brain and other central nervous system tumors and 81 % of malignancies [1]. They encompass a heterogeneous group of tumors, such as astrocytomas, oligodendrogliomas, and glioblastomas, each with varying degrees of malignancy and clinical outcomes [1]. Despite advances in therapeutic strategies, the prognosis for patients with high-grade gliomas remains poor, with a median overall survival of approximately 3 years for grade 3 gliomas and 15 months for grade 4 gliomas [2]. Maximal resection is a cornerstone in the management of gliomas, aiming to remove as much tumor tissue as possible while preserving neurological function [2]. Numerous studies have demonstrated that the extent of resection correlates positively with overall survival and progression-free survival in glioma patients [3], [4], [5]. However, achieving an optimal balance between extensive tumor removal and the avoidance of postoperative deficits poses a significant surgical challenge, especially when tumors are located in or near eloquent brain areas responsible for critical functions such as motor control, language, and sensory processing [6].

Preoperative functional mapping has become an essential tool in neurosurgical planning to identify and preserve brain eloquent regions. Traditional methods include functional magnetic resonance imaging (fMRI) and diffusion tensor imaging (DTI), which provide valuable insights into eloquent cortices and important white matter tracts [7], 8]. However, fMRI does not measure electrophysiological function, but rather metabolic increase as a substitute parameter for neural activation, and may result in false negative or false positive results due to factors such as neurovascular decoupling, magnetic susceptibility artifacts, and venous malformations [9]. Although DTI can provide important white matter bundle information, it relies on the diffusion characteristics of water molecules in the white matter, and this method may be affected by the heterogeneity and hydration of tumor tissue in tumor or lesion areas, thereby affecting its accuracy [10]. In recent years, navigated transcranial magnetic stimulation (nTMS) has emerged as a non-invasive modality for preoperative functional mapping of the cerebral cortex [11]. By stimulating specific brain regions and recording the resulting motor evoked potentials, nTMS can accurately locate functional areas such as the motor cortex or language regions. Studies have shown that nTMS can influence surgical planning by altering the intended approach or extent of resection, potentially improving surgical outcomes [12]. Several observational studies have explored the utility of nTMS in brain surgery. For instance, Krieg et al. [9] demonstrated that nTMS mapping was associated with reduced postoperative motor deficits. Similarly, Picht et al. [13] reported that nTMS provided reliable localization of motor cortex areas, which is highly consistent with intraoperative direct cortical stimulation (DCS) findings. In addition, nTMS can be combined with other neuroimaging techniques to provide reliable reconstruction of the brain’s eloquence network [14]. Meta analysis can provide more robust evaluations, increase the ability to detect significant effects, and provide more universal conclusions by statistically merging data from multiple studies. At present, most meta-analyses have analyzed the potential of nTMS in surgical management of patients with motor-eloquent brain tumors, but have been limited by small sample sizes, low quality of literature, and lack of quantitative synthesis [15], 16]. In order to clarify the role of nTMS in glioma patients, this study aims to evaluate the impact of nTMS on surgical outcomes in patients undergoing glioma resection, and assess whether nTMS can help increase resection range and reduce postoperative functional defects.

## Methods

### Literature search

A comprehensive literature search was conducted across multiple databases, including PubMed, Embase, Cochrane Library, and Web of Science, from the inception of the databases until September 26, 2024. The search strategy utilized keywords related to “navigated transcranial magnetic stimulation”, “glioma,” and “surgery,” as well as various combinations thereof. Additionally, relevant Medical Subject Headings (MeSH) terms and Emtree terms were incorporated to enhance the search coverage. This meta-analysis was conducted in accordance with the Preferred Reporting Items for Systematic Reviews and Meta-Analyses (PRISMA) guidelines, and was registered at the International Prospective Register of Systematic Reviews (PROSPERO) (https://www.crd.york.ac.uk/PROSPERO/, registration number CRD42025641788). Ethics approval and informed consent were not required because this systematic review and meta-analysis was based exclusively on previously published data and did not involve any new studies with human participants or animals performed by any of the authors.

### Study selection

The study selection process was conducted by two independent authors (Zhipeng Xu and Jinrong Wang), with any discrepancies resolved through discussion or consultation with Wenming Wang. The inclusion criteria were as follows: 1) adult patients (age ≥18 years) diagnosed with glioma; 2) Patients in the intervention group received nTMS, while patients in the control group did not receive nTMS; 3) studies that provided surgical or postoperative related outcomes; 4) case-control studies or cohort studies. Studies were excluded if they were duplicates, non-clinical research (such as reviews, expert opinions, case reports, conference abstracts, and animal studies), inaccessible full texts, or if they were not published in English.

### Quality assessment of included studies

The quality of the included studies was evaluated using the Newcastle-Ottawa Scale (NOS). The NOS assesses three main domains: selection, comparability, and exposure (or outcome). Each study can be awarded a maximum of 9 points, with higher scores indicating better methodological quality.

### Data extraction

Data extraction was performed systematically by two independent authors (Zhipeng Xu and Jinrong Wang) to gather the following information. Basic characteristics included first author, publication year, study design, country of origin, sample size, mean age, tumor site and histology. Outcome data included the extent of resection, assessments of gross motor function, postoperative complications, and quality of life evaluations.

### Data analysis

Statistical analysis was performed using RevMan 5.3. For binary outcomes, odds ratios (OR) were computed with corresponding 95 % confidence intervals (CIs). The heterogeneity among studies was evaluated using the I^2^ statistic, where I^2^ values above 50 % indicated significant heterogeneity. In cases of high heterogeneity, random-effects model was applied to account for variability among studies. Publication bias was evaluated using funnel plots due to the limited number of included studies.

**Research ethics:** Not applicable. This systematic review and meta-analysis was based exclusively on previously published data and did not involve any new studies with human participants or animals performed by any of the authors.

**Informed consent:** Not Applicable. This systematic review and meta-analysis used only previously published aggregate data and did not involve identifiable individual participant data or new participant recruitment.

## Results

**Figure 1:**
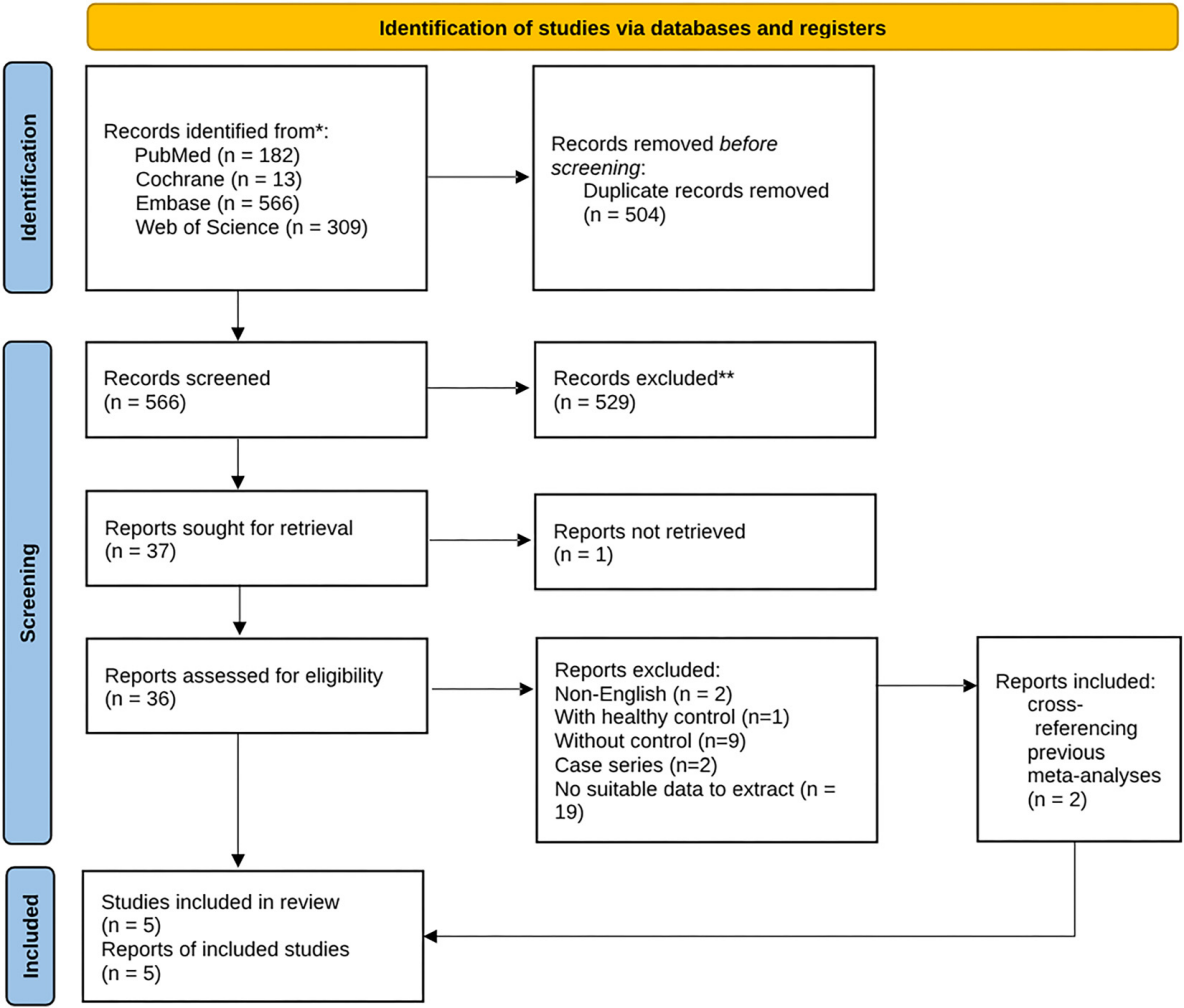
Flowchart of literature screening.

The literature screening process of this study is shown in Figure 1. The study initially retrieved 1070 articles, and finally included 5 studies by excluding and manually adding other relevant articles [17], [18], [19], [20], [21]. These 5 articles were mainly published in Europe (Germany and Italy), with a large sample size range (26–255). The age of patients varied across studies, with the median age mainly ranging from 50 to 60 years. The location of the tumor involved functionally critical areas, including the motor cortex, corticospinal tract, frontal lobe, parietal lobe, and temporal lobe (Table 1).

**Table 1:** Characteristics of included studies.

Author, publish year	Country	Study design	Group (sample size)	Age (years)^a^	Histologies	Tumor location
Frey et al. [17]	Germany	Prospective	nTMS (n=250) Control (n=115)	**nTMS:** 54 (19–82) **Control:** 53 (23–81)	**nTMS:** Glioma III/IV: 90 (36 %) Glioma II: 38 (15 %) Metastases: 85 (34 %) Other: 37 (15 %) **Control:** Glioma III/IV: 37 (32 %) Glioma II: 18 (16 %) Metastases: 40 (35 %) Other: 20 (17 %)	**nTMS:** Motor cortex: 88 (35 %) Corticospinal tract: 35 (14 %) Both: 127 (51 %) **Control:** Motor cortex: 32 (28 %) Corticospinal tract: 22 (19 %) Both: 61 (53 %)
Raffa et al. [16]	Italy	Retrospective	nTMS+SFG+IONM (n=41) IONM (n=41)	**nTMS+SFG+IONM:** 59.27 ± 14.15 **IONM**: 56.49 ± 12.53	**nTMS++SFG+ IONM:** Glioblastomas: 31 Anaplastic astrocytomas: 10 **IONM:** Glioblastomas: 28 Anaplastic astrocytomas: 13	**nTMS+SFG+ IONM:** Frontal: 31 Parietal: 18 Temporal: 13 Insular: 6 **IONM:** Frontal: 28 Parietal: 17 Temporal: 14 Insular: 6
Raffa et al. [19]	Italy	Prospective	nTMS+SFG+IONM (n=79) IONM (n=55)	**nTMS+SFG+IONM:** 56.4 ± 13.7 **IONM:** 56.5 ± 12.3	**nTMS+SFG+IONM:** Glioblastoma: 61 Anaplastic astrocytoma: 15 Anaplastic oligodendrogliomas: 3 **IONM:** Glioblastoma: 42 Anaplastic astrocytoma: 11 Anaplastic oligodendrogliomas: 2	**/**
Hendrix et al. [18]	Germany	Retrospective	nTMS (n=47) Non-nTMS (n=47)	**nTMS:** 162.4 ± 15.2 **Non-nTMS:** 58.1 ± 12.1	Malignant gliomas (WHO III and IV)	**nTMS:** Frontal: 33 Parietal: 14 **Non-nTMS:** Frontal: 33 Parietal: 14
Weiss Lucas et al. [21]	Germany	Prospective	nTMS (n=35) Control (n=26)	63 (32–87)	IDH1 R132H wildtype, immunohistochemical analysis: 55 (90 %) IDH1/IDH 2 wildtype, moleculargenetic analysis: 39 (63 %) Wildtype unknown 5 (8 %)	Frontal and/or parietal localisation, and partly involved the temporal lobe (25 %), and/or reached the insula (33 %), and/or the sylvian fissure (12 %)

nTMS, navigated transcranial magnetic stimulation; DTI, diffusion tensor imaging; SFG, sodium fluoresceine-guided; IONM, intraoperative neurophysiologic mapping. ^a^Age was presented in mean ± standard deviation or median (range). Bold values indicate the intervention or comparator group labels within each included study.

### Quality assessment

Overall, the quality of the included literature was good, and the scores of all articles were above 7 points. Among them, Frey et al. scored the highest, with a quality score of 9 points, reflecting the rigor of the methodology (Table 2).

**Table 2:** Quality assessment of studies.

Study	Representativeness of the exposed cohort	Selection of the non-exposed cohort	Ascertainment of exposure	Demonstration that outcome of interest was not present at start of study	Comparability of cohorts on the basis of the design or analysis	Assessment of outcome	Was follow-up long enough for outcomes to occur	Adequacy of follow up of cohorts	Quality score
Frey et al. [17]	a	a	a	a	b	a	a	a	9
Raffa et al. [16]	a	a	a		a	a	a	a	7
Raffa et al. [19]	a	a	a		b	a	a	a	8
Hendrix et al. [18]	a	a	a		b	a	a	a	8
Weiss Lucas et al. [21]	a		a	a	a	a	a	a	7

a, one star was awarded for the corresponding Newcastle–Ottawa Scale item; b, two stars were awarded for the corresponding Newcastle–Ottawa Scale item.

### nTMS group vs. non-nTMS group

Three studies compared the extent of tumor resection, gross motor function, quality of life, and postoperative complications between the nTMS group and the non-nTMS group [17], 18], 21].

#### Extent of resection

**Figure 2:**
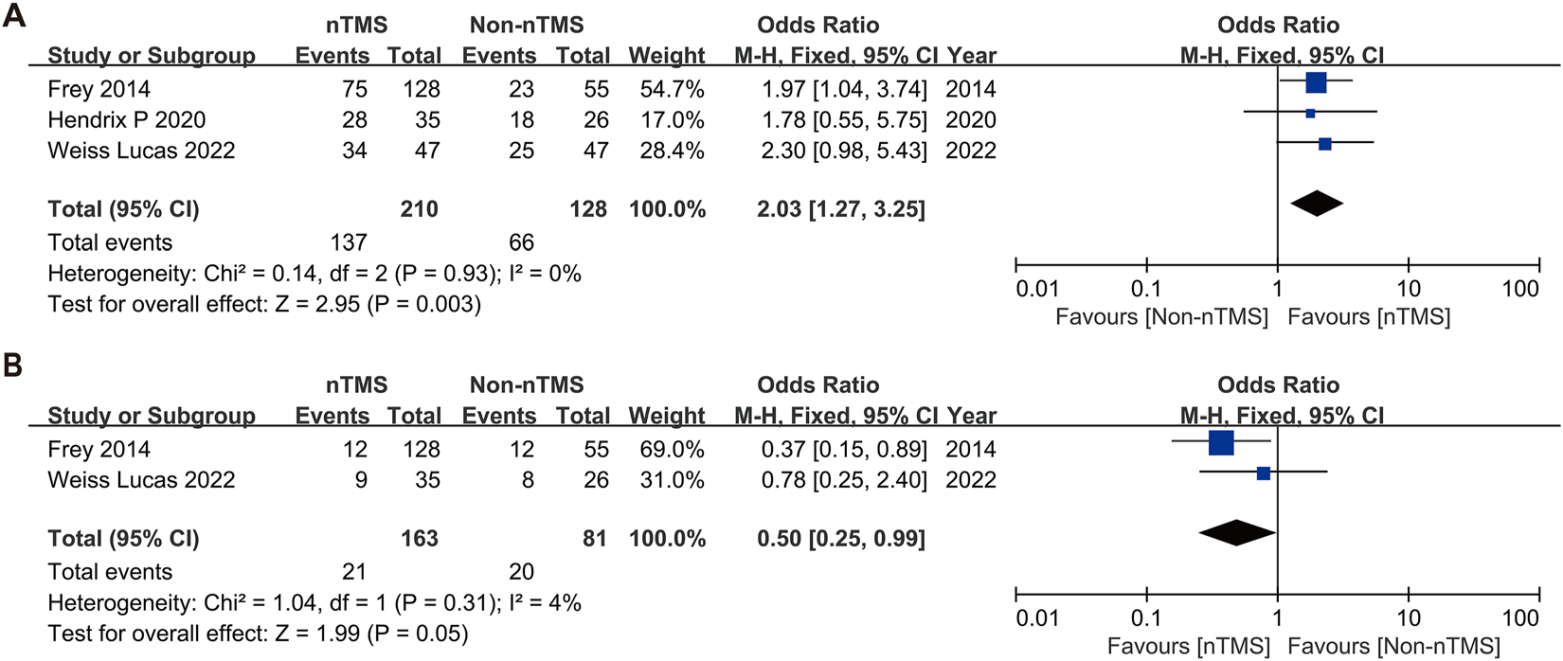
Comparison of different degrees of glioma resection in patients in the nTMS group and the non-nTMS group. A: Gross total resection; B: subtotal resection.

As shown in Figure 2A, the gross total resection (GTR) rate was significantly higher in the nTMS group compared with the non-nTMS group (OR=2.03; 95 % CI: 1.27–3.25; p=0.003), and the heterogeneity among the 3 studies was low. For subtotal resection (STR), the nTMS group had a lower STR rate compared with the non-nTMS group (OR=0.50; 95 % CI: 0.25–0.99; p=0.05), with an I^2^ of 4 % (Figure 2B). These results suggested that nTMS mapping improves surgical precision.

#### Gross motor function

**Figure 3:**
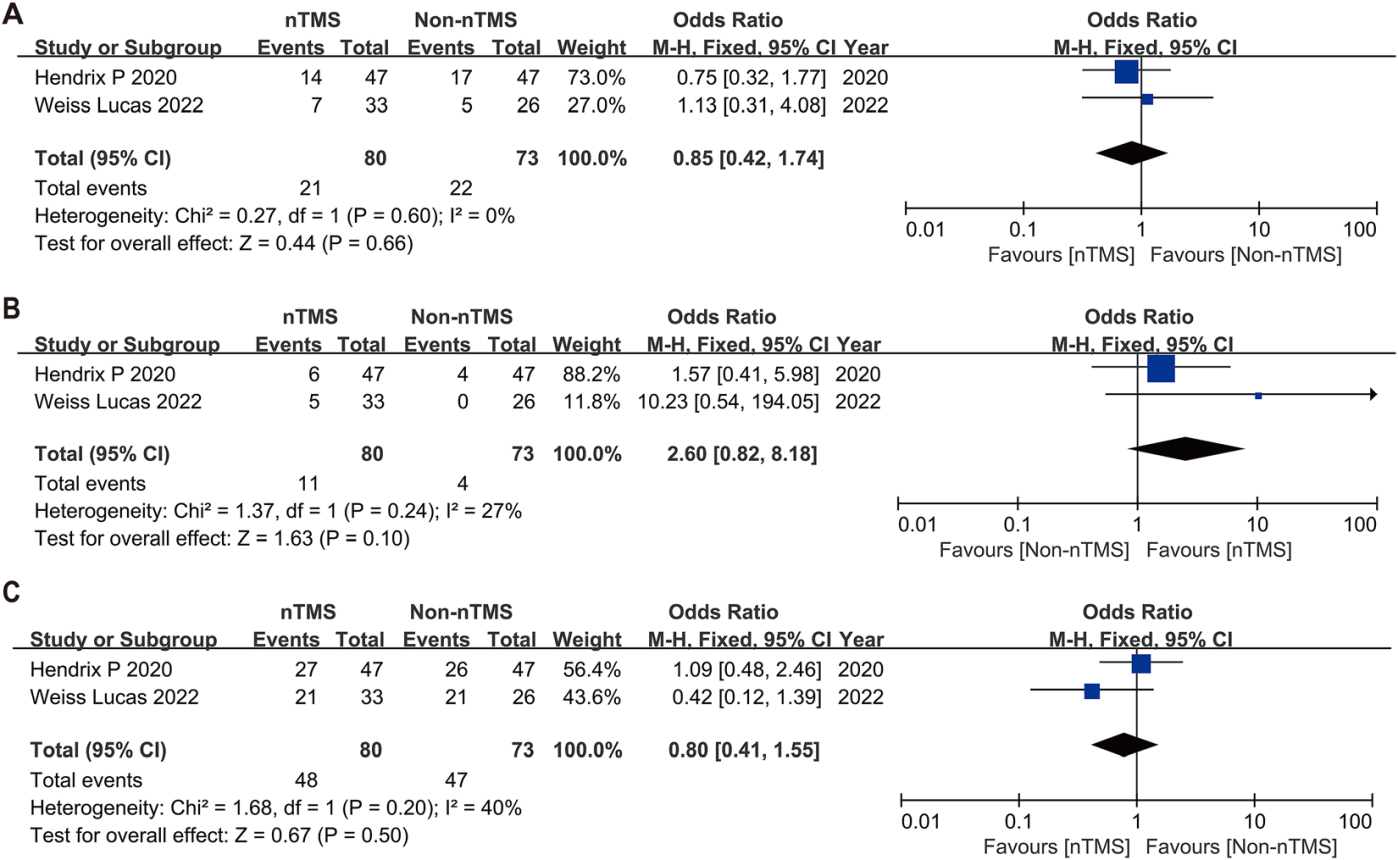
Comparison of gross motor function between the nTMS group and the non-nTMS group after surgery. A: Patients’ gross motor function improved after surgery; B: patients’ gross motor function deteriorated after surgery; C: patients’ gross motor function unchanged after surgery.

In terms of postoperative motor function, nTMS did not appear to demonstrate an advantage compared with non-nTMS. When comparing patients with different grades of postoperative gross motor function, although component heterogeneity was good. However, no statistical differences were found between the two groups, whether they were classified as improved (OR=0.85; 95 % CI: 0.42–1.74; p=0.66) (Figure 3A), worsened (OR=2.60; 95 % CI: 0.82–8.18; p=0.10) (Figure 3B), or unchanged (OR=0.80; 95 % CI: 0.41–1.55; p=0.50) (Figure 3C) in postoperative gross motor function.

#### Quality of life

**Figure 4:**
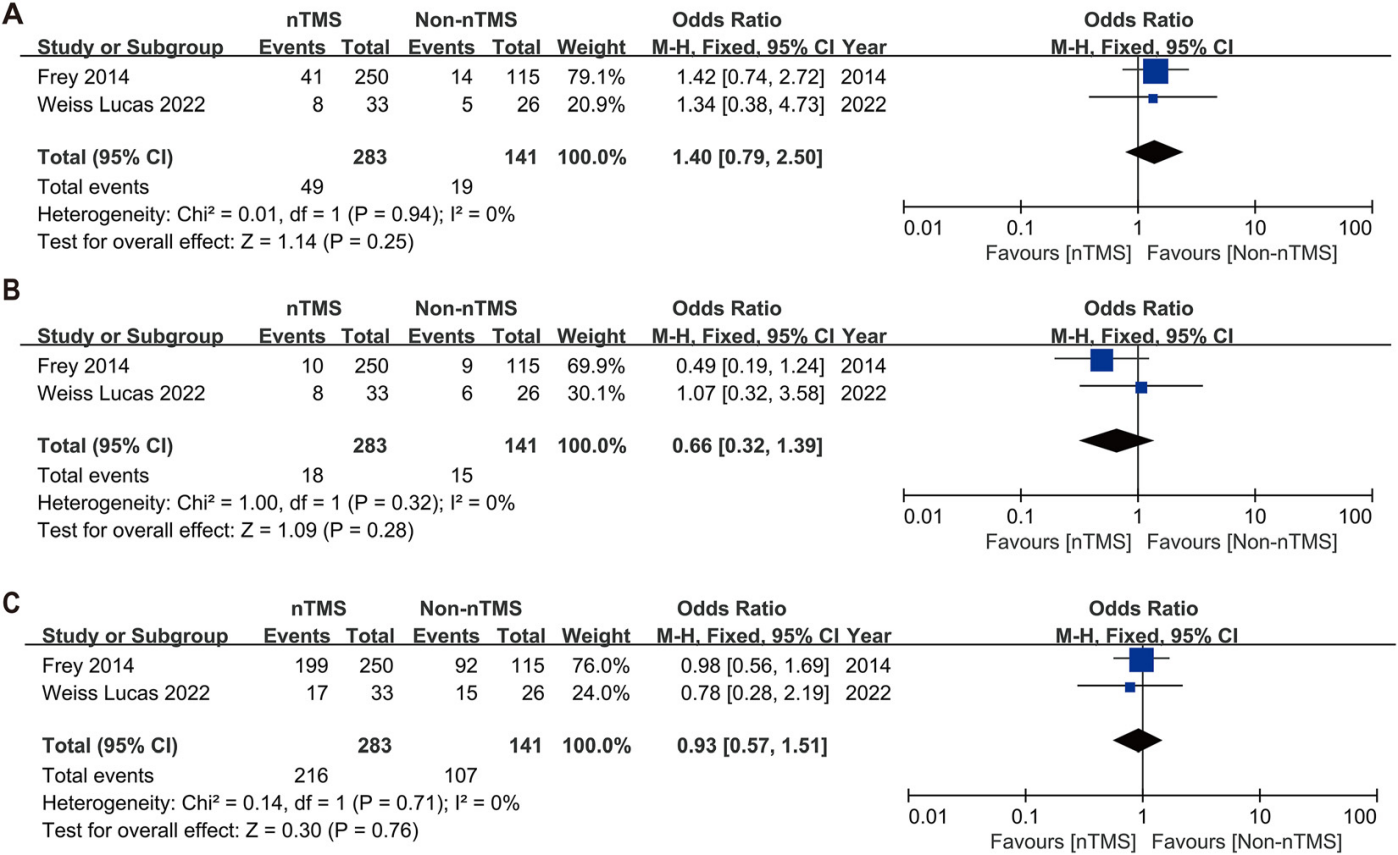
Comparison of Karnofsky performance status between the nTMS group and the non-nTMS group after surgery. A: Patients’ quality of life improved after surgery; B: patients’ quality of life deteriorated after surgery; C: patients’ quality of life unchanged after surgery.

The quality of life assessed by Karnofsky performance status (KPS) was shown in Figure 4. There was no significant increase in the number of patients with improved KPS after surgery in the nTMS group (OR=1.40; 95 % CI: 0.79–2.50; p=0.25) (Figure 4A). Relatively fewer patients in the nTMS group had a decrease in KPS score compared with the non-nTMS group, but there was no significant difference between the groups (OR=0.66; 95 % CI: 0.32–1.39; p=0.28) (Figure 4B). The number of patients with no change in KPS was also similar in the two groups (OR=0.93; 95 % CI: 0.57–1.51; p=0.76) (Figure 4C). This suggested that nTMS had no significant effect on maintaining a higher quality of life after surgery.

#### Postoperative complications

The difference in postoperative complications between the two groups was reported in one study, with the results showing that 4.3 % of patients in both the nTMS and non-nTMS groups experienced surgery-related transient paralysis, while the incidence of permanent paralysis in the nTMS group (6/47) was slightly higher than that in the non-nTMS group (4/47), although there was no significant difference between the groups [18].

### nTMS+SFG+IONM group vs. IONM group

Two studies compared the extent of tumor resection and gross motor function in patients treated with nTMS+ subcortical fiber tracking (SFG)+ intraoperative neurophysiological monitoring (IONM) and IONM [19], 20].

#### Extent of resection

In both two studies, compared to patients in the IONM group, those in the nTMS+SFG+IONM group demonstrated a significantly higher rate of GTR (p<0.05). Although the rate of STR was lower in the nTMS+SFG+IONM group, the difference was not statistically significant compared to the IONM group (p>0.05) [19], 20].

#### Gross motor function

One study reported that, in terms of postoperative motor function preservation, patients in the nTMS+SFG+IONM group had significantly higher Medical Research Council scores than those in the IONM group (p<0.05), with a greater proportion of patients showing postoperative motor improvement [20]. Although new permanent motor deficits were observed in both groups, the incidence was significantly lower in the nTMS+SFG+IONM group (p=0.04), and these patients also had higher Karnofsky Performance Status scores (p=0.03). Similar findings were observed in another study, although without statistical significance [19].

### Risk of bias

Funnel plots were further used to assess the potential for publication bias. The results showed that all funnel plots exhibited good symmetry with no apparent asymmetry, suggesting the absence of significant publication bias (Figure S1).

## Discussion

The main result of the present meta-analysis evaluated potential of nTMS as an adjunctive tool in glioma surgery. The analysis highlights that nTMS appears to contribute positively to surgical outcomes, notably by increasing the extent of tumor resection while providing a comparable safety profile in terms of postoperative neurological complications.

Our analysis indicates that nTMS significantly enhances surgical outcomes by increasing the extent of tumor resection without compromising postoperative neurological safety. Specifically, patients in the nTMS group exhibited higher rates of GTR and lower rates of STR compared to those who did not receive nTMS mapping, effectively doubling the odds of achieving GTR. This increase in GTR is clinically significant, as previous studies have demonstrated that patients undergoing GTR have significantly reduced mortality rates at 1 and 2 years postoperatively compared with those undergoing STR, underscoring the importance of maximal safe resection in enhancing survival [4]. The enhanced GTR rate reflects nTMS’s utility in guiding surgical planning by providing detailed cortical mapping, which is critical for determining resection boundaries and minimizing the risk of functional damage. For instance, Picht et al. reported that preoperative mapping with nTMS in motor-eloquent areas was associated with lower rates of residual tumor on postoperative imaging, a decrease in surgery-related paresis, and reduced craniotomy size, suggesting that nTMS can significantly enhance surgical precision while minimizing invasiveness [22].

However, the results indicate that nTMS has a limited effect on improving postoperative KPS or reducing postoperative complications rates. The absence of significant improvement may be attributed to the complexity of glioma cases. Surgical complications themselves, particularly hemorrhagic events, are predictive of poorer functional outcomes and lower postoperative KPS scores in patients with motor pathway involvement [23]. Moreover, quality of life outcomes in glioma patients are significantly influenced by factors such as tumor grade, extent of resection, and preoperative health status [24]. Besides, while nTMS is effective for cortical mapping, it may not adequately address subcortical pathways that are critical for maintaining neurological function. Studies have shown that intraoperative mapping of subcortical white matter tracts during glioma resection significantly reduces postoperative morbidity by preserving critical neural pathways [25]. Combining nTMS with intraoperative subcortical mapping and neuromonitoring may thus offer a more comprehensive approach for optimizing functional outcomes in glioma surgeries.

Two studies comparing a multimodal strategy (nTMS + SFG + IONM) with IONM alone reported higher GTR rates and lower rates of permanent motor deficit in the multimodal arm. Although the STR rate was not significantly different between groups, the numerical reduction further supports the value of integrating pre-operative and intra-operative technologies. In a study, patients with language-eloquent left-hemispheric gliomas underwent preoperative nTMS-based language mapping and DTI tractography, followed by awake craniotomy using direct electrical stimulation and IONM, the multimodal approach resulted was effective in reducing the incidence of permanent deficits and maintaining functional status, as measured by the KPS scores postoperatively, highlighting the benefit of combining preoperative nTMS mapping with intraoperative functional monitoring for optimal resection in eloquent regions [26]. This combination improved resection safety and helped preserve language functions in highly eloquent areas, demonstrating the value of this advanced setup in glioma surgeries involving critical networks. Besides, when nTMS was combined with SFG resection and IONM, patients exhibited better postoperative motor function compared to those who underwent IONM alone. While nTMS alone did not show a significant advantage in postoperative motor function. Even though only one of the two reached statistical significance for functional endpoints [20]. This finding may highlight the limitations of relying on preoperative mapping alone to prevent postoperative motor deficits, as intraoperative factors like dynamic shifts in brain anatomy due to tissue manipulation, cerebrospinal fluid changes, and gravitational effects significantly influence surgical outcomes. Therefore, integrating preoperative nTMS with IONM and other intraoperative tools has been shown to be advantageous in preserving motor function during glioma surgeries [15]. Because both multimodal studies (nTMS + SFG + IONM vs. IONM alone) were conducted by the same research group (Raffa et al.), we chose not to pool their data to avoid a potential researcher effect. Additional independent, controlled studies are therefore urgently required to validate these findings and to permit a robust quantitative synthesis in future meta-analyses.

Nonetheless, there are limitations to consider in our study. The number of studies included was small, and the majority were conducted in Europe, potentially limiting the generalizability of the findings to other populations. Additionally, the heterogeneity in study designs, patient characteristics, and outcome measures may have affected the robustness of the meta-analysis. The limited number of studies also precluded a comprehensive assessment of publication bias. Future research should focus on large-scale, multicenter randomized controlled trials to further elucidate the role of nTMS in glioma surgery.

## Conclusions

This meta-analysis indicates that pre-operative nTMS mapping substantially increases gross-total resection rates compared with conventional planning. Its combination with SFG-based tractography and IONM may further enhance anatomical and functional outcomes, although current supporting evidence is limited. While early postoperative motor and quality-of-life benefits remain inconclusive, the oncological gains and absence of excess morbidity justify the integration of nTMS, alone or within a multimodal workflow, into contemporary surgical management of motor-eloquent gliomas. Further large-scale studies are warranted to validate these results and establish standardized protocols for the use of nTMS in neurosurgical oncology.

## Supplementary Material

Supplementary Material

Supplementary Material
