# Supplementary figures and images for: Effect of navigated transcranial magnetic stimulation for glioma surgery outcomes: a systematic review and meta-analysis
Source: Open Med (Wars). 2026 Jul 2;21(1):20251326. doi: 10.1515/med-2025-1326 (PMC13321228; doi:10.1515/med-2025-1326)

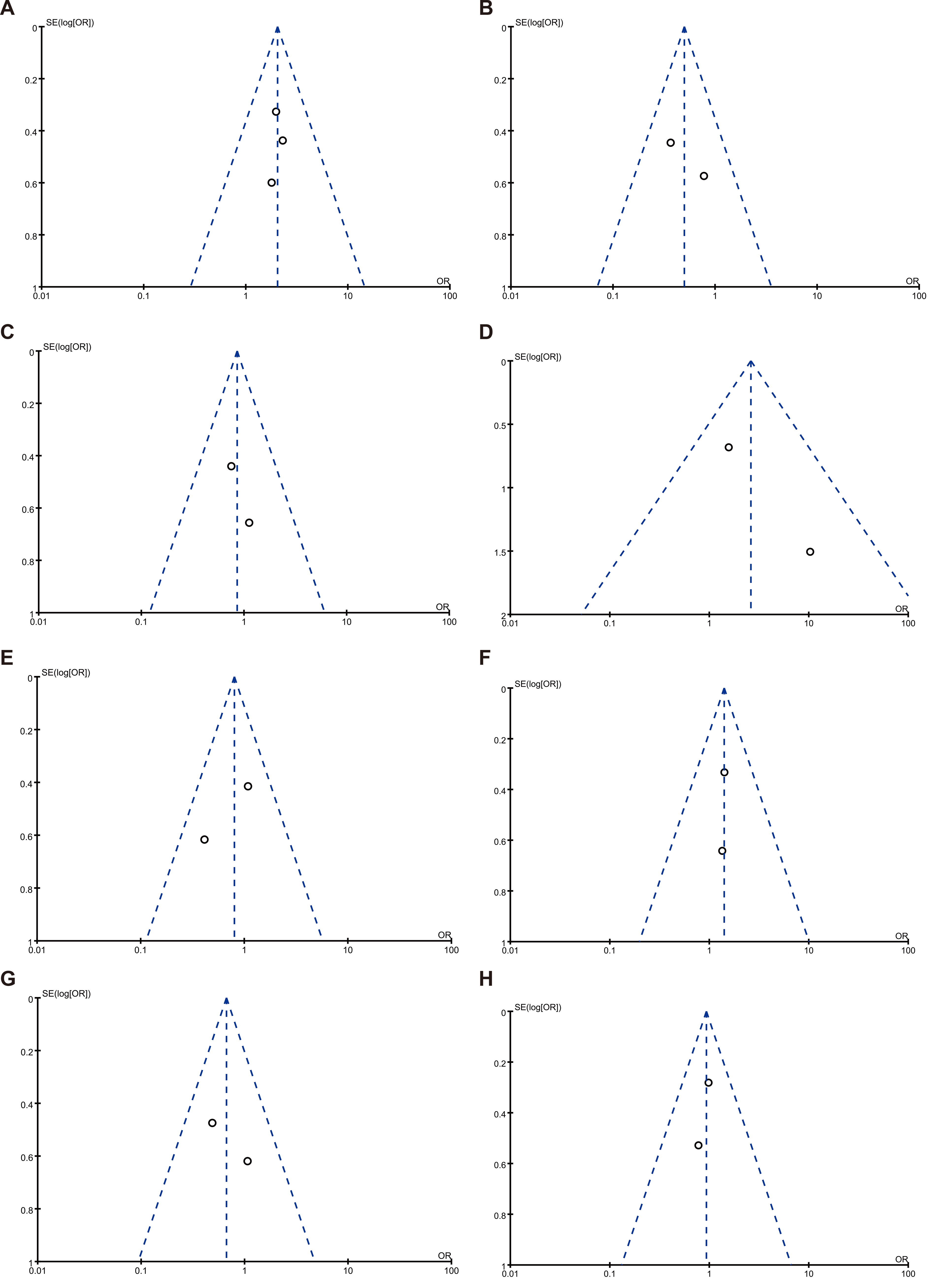

Supplement: Supplementary file 1 — Supplementary Material [file j_med-2025-1326_suppl_001.jpg]
