# Supplementary material for: Effect of navigated transcranial magnetic stimulation for glioma surgery outcomes: a systematic review and meta-analysis
Source: Open Med (Wars). 2026 Jul 2;21(1):20251326. doi: 10.1515/med-2025-1326 (PMC13321228; doi:10.1515/med-2025-1326)
Supplement: Supplementary file 2 — Supplementary Material [file j_med-2025-1326_suppl_002.docx]

Supplementary Figure 1. Funnel plot between nTMS group and non-nTMS group. A: Gross total resection; B: Subtotal resection; C: Patients' gross motor function improved after surgery; D: Patients' gross motor function deteriorated after surgery; E: Patients' gross motor function unchanged after surgery; F: Patients' quality of life improved after surgery; G: Patients' quality of life deteriorated after surgery; H: Patients' quality of life unchanged after surgery.
